# Supplementary figures and images for: Metabolite Production in Alkanna tinctoria Links Plant Development with the Recruitment of Individual Members of Microbiome Thriving at the Root-Soil Interface
Source: mSystems. 2022 Sep 7;7(5):e00451-22. doi: 10.1128/msystems.00451-22 (PMC9601132; doi:10.1128/msystems.00451-22)

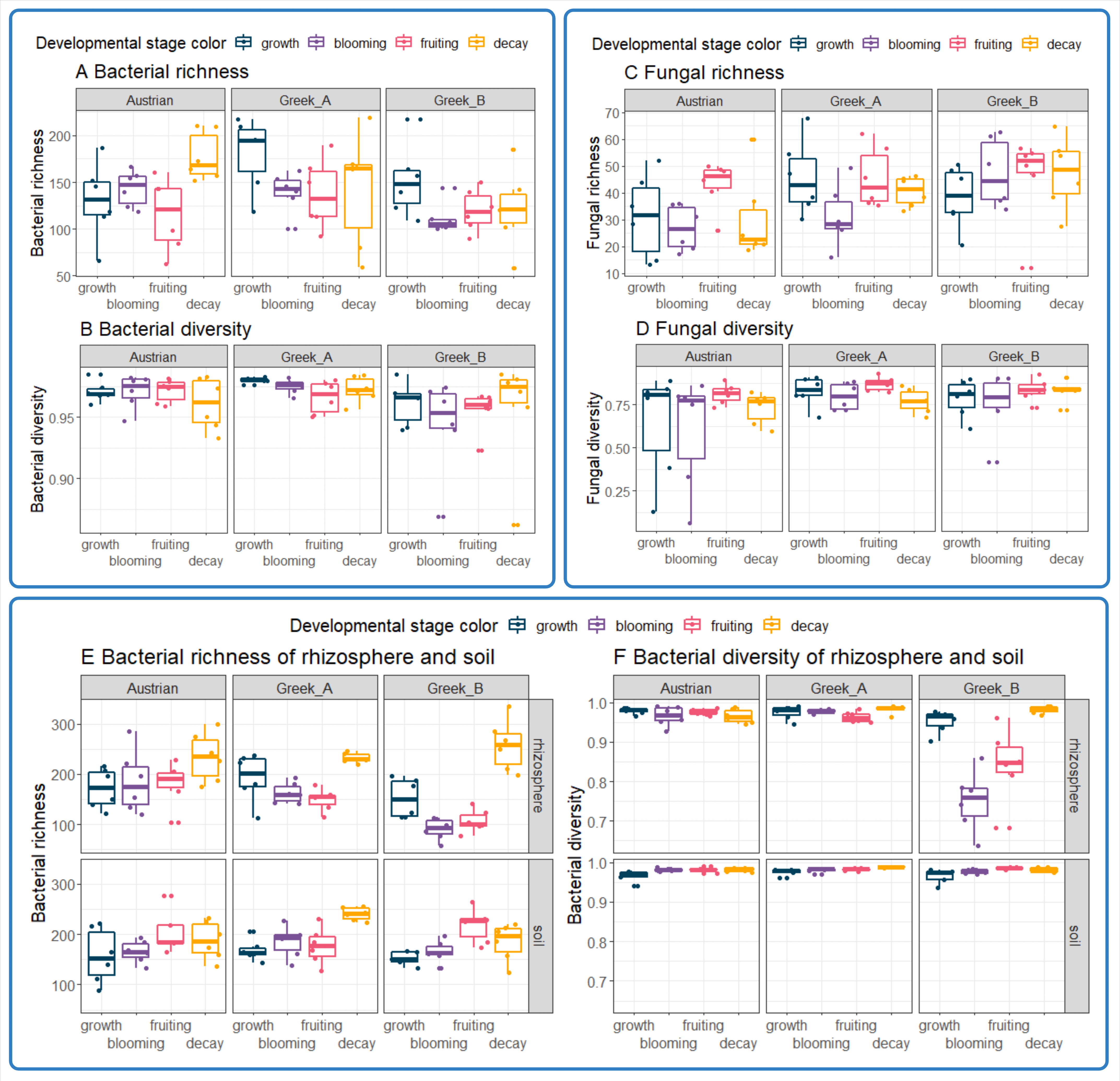

Supplement: FIG S1 [file msystems.00451-22-s0001.jpg]

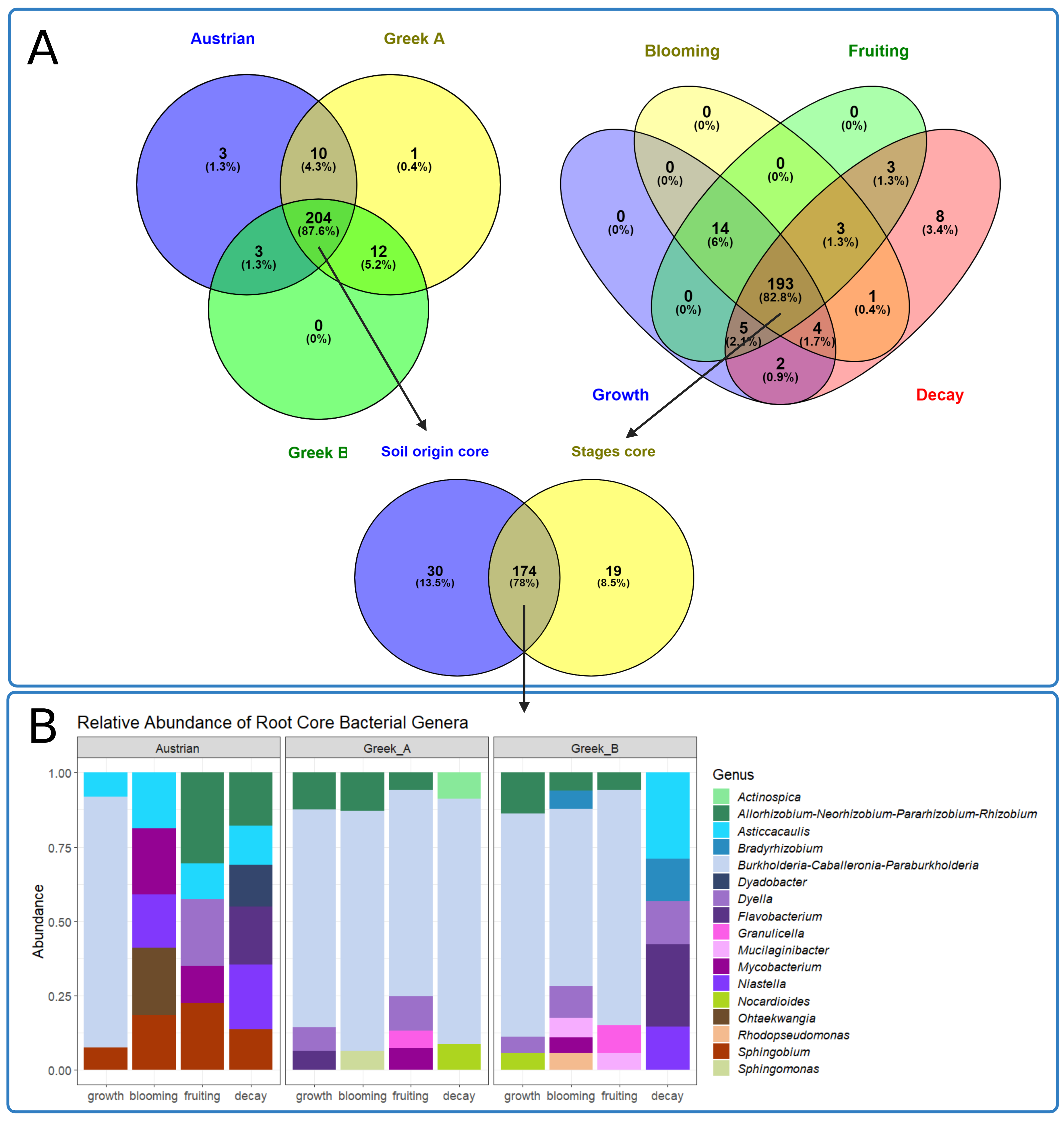

Supplement: FIG S2 [file msystems.00451-22-s0002.tif]

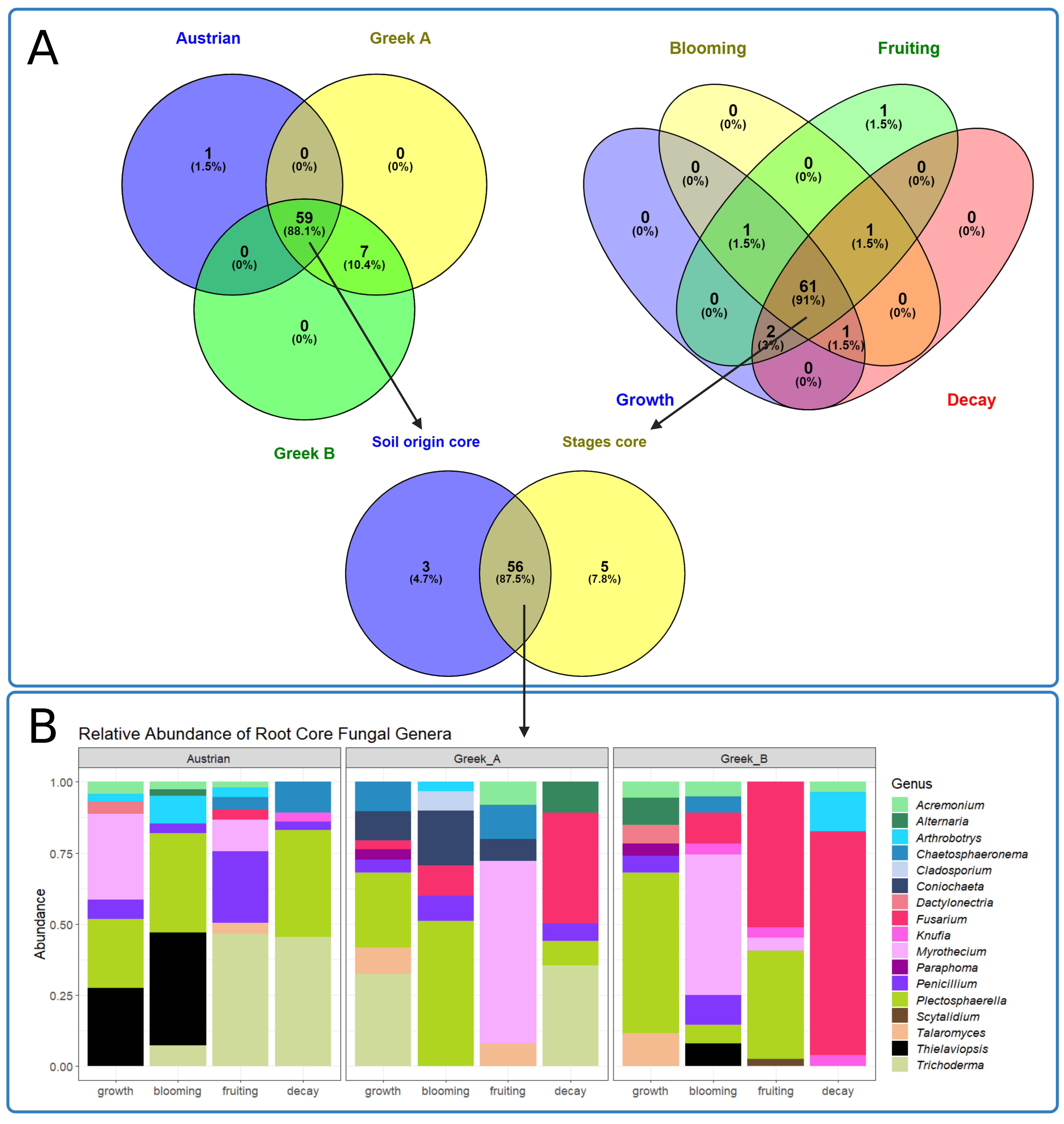

Supplement: FIG S3 [file msystems.00451-22-s0003.tif]

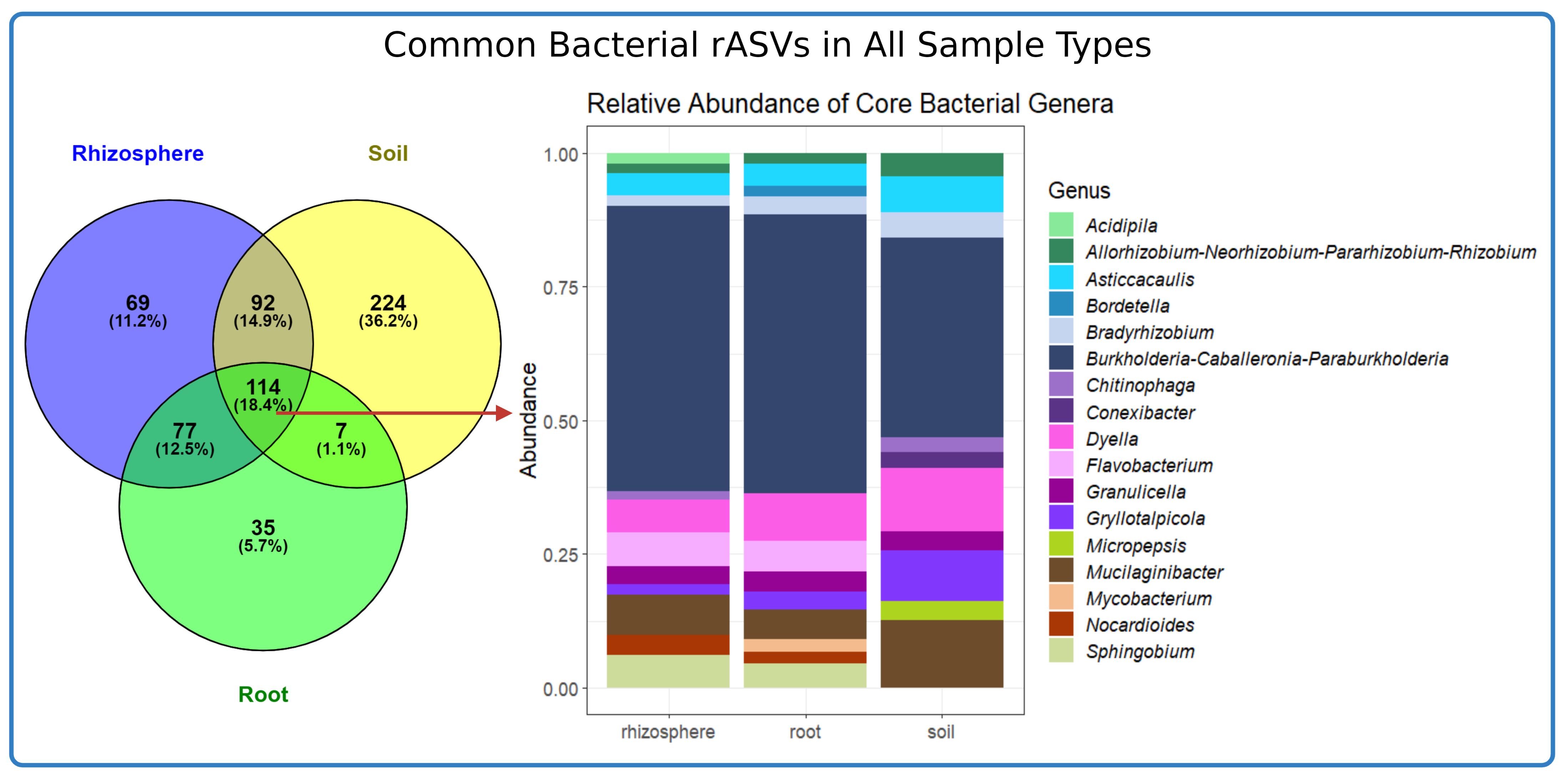

Supplement: FIG S4 [file msystems.00451-22-s0004.tif]

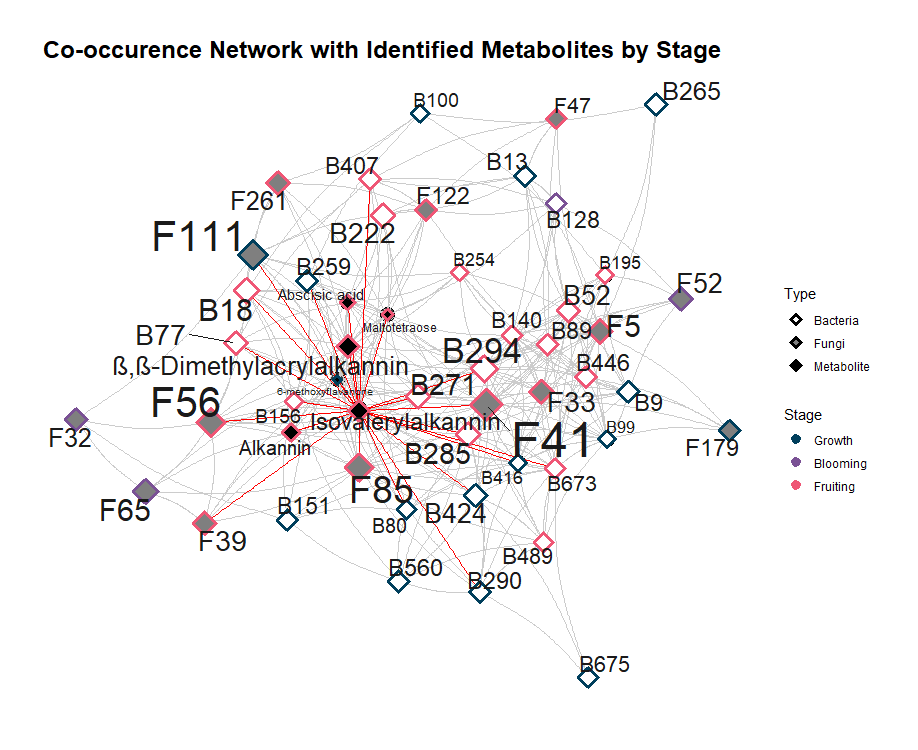

Supplement: FIG S5 [file msystems.00451-22-s0005.tif]
