# Supplementary material for: Metabolite Production in Alkanna tinctoria Links Plant Development with the Recruitment of Individual Members of Microbiome Thriving at the Root-Soil Interface
Source: mSystems. 2022 Sep 7;7(5):e00451-22. doi: 10.1128/msystems.00451-22 (PMC9601132; doi:10.1128/msystems.00451-22)
Supplement: TABLE S1 [file msystems.00451-22-s0006.docx]

| **Bacteria** | | | | **Fungi** | | | |
| --- | --- | --- | --- | --- | --- | --- | --- |
| **Austrian** | | | | **Austrian** | | | |
| Pairs | F mod. | *R*^2^ | p value | Pairs | F mod. | *R*^2^ | p value |
| Growth vs Blooming | 1.08 | 0.10 | 0.322 | Growth vs Blooming | 0.89 | 0.08 | 0.691 |
| Blooming vs Fruiting | 1.26 | 0.11 | 0.282 | Blooming vs Fruiting | 2.04 | 0.17 | 0.072 |
| Fruiting vs Decay | 1.83 | 0.15 | **0.008**** | Fruiting vs Decay | 1.53 | 0.13 | 0.184 |
| **Greek A** | | | | **Greek A** | | | |
| Pairs | F mod. | *R*^2^ | p value | Pairs | F mod. | *R*^2^ | p value |
| Growth vs Blooming | 1.32 | 0.12 | 0.109 | Growth vs Blooming | 0.88 | 0.08 | 0.650 |
| Blooming vs Fruiting | 1.03 | 0.09 | 0.423 | Blooming vs Fruiting | 1.41 | 0.12 | 0.140 |
| Fruiting vs Decay | 2.42 | 0.19 | **0.022*** | Fruiting vs Decay | 1.37 | 0.12 | 0.140 |
| **Greek B** | | | | **Greek B** | | | |
| Pairs | F mod. | *R*^2^ | p value | Pairs | F mod. | *R*^2^ | p value |
| Growth vs Blooming | 1.68 | 0.14 | 0.064 | Growth vs Blooming | 1.14 | 0.10 | 0.288 |
| Blooming vs Fruiting | 1.47 | 0.13 | 0.064 | Blooming vs Fruiting | 2.55 | 0.20 | **0.027*** |
| Fruiting vs Decay | 3.84 | 0.28 | **0.006**** | Fruiting vs Decay | 1.41 | 0.12 | 0.100 |
| **Growth stage** | | | | **Growth stage** | | | |
| Pairs | F mod. | *R*^2^ | p value | Pairs | F mod. | *R*^2^ | p value |
| Greek A vs Greek B | 1.44 | 0.13 | 0.115 | Greek A vs Greek B | 0.85 | 0.08 | 0.865 |
| Greek A vs Austrian | 1.89 | 0.16 | 0.054 | Greek A vs Austrian | 0.75 | 0.07 | 0.865 |
| Greek B vs Austrian | 1.35 | 0.12 | 0.115 | Greek B vs Austrian | 0.82 | 0.08 | 0.865 |
| **Blooming stage** | | | | **Blooming stage** | | | |
| Pairs | F mod. | *R*^2^ | p value | Pairs | F mod. | *R*^2^ | p value |
| Greek A vs Greek B | 4.29 | 0.30 | **0.006**** | Greek A vs Greek B | 2.19 | 0.18 | **0.021*** |
| Greek A vs Austrian | 3.48 | 0.26 | **0.006**** | Greek A vs Austrian | 1.59 | 0.14 | **0.021*** |
| Greek B vs Austrian | 4.34 | 0.30 | **0.006**** | Greek B vs Austrian | 1.96 | 0.16 | **0.021*** |
| **Fruiting stage** | | | | **Fruiting stage** | | | |
| Pairs | F mod. | *R*^2^ | p value | Pairs | F mod. | *R*^2^ | p value |
| Greek A vs Greek B | 3.20 | 0.24 | **0.009**** | Greek A vs Greek B | 2.06 | 0.17 | **0.012*** |
| Greek A vs Austrian | 2.02 | 0.17 | 0.074 | Greek A vs Austrian | 1.63 | 0.14 | **0.048*** |
| Greek B vs Austrian | 4.43 | 0.31 | **0.003**** | Greek B vs Austrian | 4.36 | 0.30 | **0.009**** |
| **Decay stage** | | | | **Decay stage** | | | |
| Pairs | F mod. | *R*^2^ | p value | Pairs | F mod. | *R*^2^ | p value |
| Greek A vs Greek B | 1.58 | 0.14 | **0.021*** | Greek A vs Greek B | 1.13 | 0.10 | 0.249 |
| Greek A vs Austrian | 2.26 | 0.18 | **0.015*** | Greek A vs Austrian | 1.68 | 0.14 | **0.039*** |
| Greek B vs Austrian | 1.75 | 0.15 | **0.021*** | Greek B vs Austrian | 2.27 | 0.18 | **0.006**** |

| **Rhizosphere** | | | | **Bulk soil** | | | |
| --- | --- | --- | --- | --- | --- | --- | --- |
| **Austrian** | | | | **Austrian** | | | |
| Pairs | F mod. | *R*^2^ | p value | Pairs | F mod. | *R*^2^ | p value |
| Growth vs Blooming | 1.652 | 0.142 | **0.009**** | Growth vs Blooming | 1.638 | 0.14 | **0.0345*** |
| Blooming vs Fruiting | 1.573 | 0.136 | **0.006**** | Blooming vs Fruiting | 0.941 | 0.095 | 0.468 |
| Fruiting vs Decay | 2.152 | 0.177 | **0.006**** | Fruiting vs Decay | 2.738 | 0.233 | **0.012*** |
| **Greek A** | | | | **Greek A** | | | |
| Pairs | F mod. | *R*^2^ | p value | Pairs | F mod. | *R*^2^ | p value |
| Growth vs Blooming | 1.716 | 0.146 | **0.026*** | Growth vs Blooming | 2.09 | 0.173 | **0.007**** |
| Blooming vs Fruiting | 1.227 | 0.109 | 0.193 | Blooming vs Fruiting | 1.963 | 0.164 | **0.012*** |
| Fruiting vs Decay | 2.362 | 0.191 | **0.006**** | Fruiting vs Decay | 3.889 | 0.28 | **0.005**** |
| **Greek B** | | | | **Greek B** | | | |
| Pairs | F mod. | *R*^2^ | p value | Pairs | F mod. | *R*^2^ | p value |
| Growth vs Blooming | 5.801 | 0.367 | **0.006**** | Growth vs Blooming | 1.496 | 0.13 | 0.053 |
| Blooming vs Fruiting | 1.741 | 0.148 | 0.097 | Blooming vs Fruiting | 1.41 | 0.124 | 0.077 |
| Fruiting vs Decay | 6.989 | 0.411 | **0.006**** | Fruiting vs Decay | 3.8 | 0.275 | **0.006*** |
| **Growth stage** | | | | **Growth stage** | | | |
| Pairs | F mod. | *R*^2^ | p value | Pairs | F mod. | *R*^2^ | p value |
| Greek A vs Greek B | 2.25 | 0.184 | **0.005**** | Greek A vs Greek B | 3.911 | 0.281 | **0.006**** |
| Greek A vs Austrian | 2.187 | 0.179 | **0.005**** | Greek A vs Austrian | 5.386 | 0.35 | **0.005**** |
| Greek B vs Austrian | 2.689 | 0.212 | **0.005**** | Greek B vs Austrian | 4.472 | 0.309 | **0.005**** |
| **Blooming stage** | | | | **Blooming stage** | | | |
| Pairs | F mod. | *R*^2^ | p value | Pairs | F mod. | *R*^2^ | p value |
| Greek A vs Greek B | 13.257 | 0.57 | **0.003**** | Greek A vs Greek B | 4.193 | 0.296 | **0.006**** |
| Greek A vs Austrian | 3.858 | 0.278 | **0.003**** | Greek A vs Austrian | 5.275 | 0.345 | **0.009**** |
| Greek B vs Austrian | 8.714 | 0.466 | **0.003**** | Greek B vs Austrian | 3.599 | 0.265 | **0.003**** |
| **Fruiting stage** | | | | **Fruiting stage** | | | |
| Pairs | F mod. | *R*^2^ | p value | Pairs | F mod. | *R*^2^ | p value |
| Greek A vs Greek B | 4.815 | 0.325 | **0.006**** | Greek A vs Greek B | 1.909 | 0.16 | **0.024*** |
| Greek A vs Austrian | 1.812 | 0.153 | 0.076 | Greek A vs Austrian | 1.371 | 0.132 | 0.132 |
| Greek B vs Austrian | 6.039 | 0.377 | **0.006**** | Greek B vs Austrian | 2.177 | 0.195 | **0.024*** |
| **Decay stage** | | | | **Decay stage** | | | |
| Pairs | F mod. | *R*^2^ | p value | Pairs | F mod. | *R*^2^ | p value |
| Greek A vs Greek B | 2.149 | 0.177 | **0.007**** | Greek A vs Greek B | 2.654 | 0.209 | **0.003**** |
| Greek A vs Austrian | 2.395 | 0.193 | **0.006**** | Greek A vs Austrian | 6.937 | 0.409 | **0.003**** |
| Greek B vs Austrian | 1.72 | 0.147 | **0.006**** | Greek B vs Austrian | 5.063 | 0.336 | **0.003**** |

^a^statistically significant p values presented in bold letters, *p<0.05, **p<0.01
