# Supplementary material for: Metabolite Production in Alkanna tinctoria Links Plant Development with the Recruitment of Individual Members of Microbiome Thriving at the Root-Soil Interface
Source: mSystems. 2022 Sep 7;7(5):e00451-22. doi: 10.1128/msystems.00451-22 (PMC9601132; doi:10.1128/msystems.00451-22)
Supplement: TABLE S2 [file msystems.00451-22-s0007.docx]

| **Soil = Austrian** | | | | | |
| --- | --- | --- | --- | --- | --- |
| Stage | emmean | SE | lower.CL | upper.CL | Contrast group |
| blooming | 31.1 | 22.8 | -27.6 | 89.8 | a |
| decay | 11.7 | 25 | -52.6 | 76 | a |
| fruiting | 198.1 | 22.8 | 139.4 | 256.8 | b |
| growth | 26 | 22.8 | -32.7 | 84.7 | a |
|  |  |  |  |  |  |
| **Soil = Greek A** | | | | | |
| Stage | emmean | SE | lower.CL | upper.CL | Contrast group |
| blooming | 20.1 | 22.8 | -38.6 | 78.8 | a |
| decay | 28.3 | 22.8 | -30.4 | 87 | a |
| fruiting | 92.3 | 22.8 | 33.6 | 151 | a |
| growth | 33.6 | 22.8 | -25.1 | 92.3 | a |
|  |  |  |  |  |  |
| **Soil = Greek B** | | | | | |
| Stage | emmean | SE | lower.CL | upper.CL | Contrast group |
| blooming | 20 | 22.8 | -38.7 | 78.7 | a |
| decay | 0 | 22.8 | -58.7 | 58.7 | a |
| fruiting | 193.6 | 22.8 | 134.9 | 252.3 | b |
| growth | 19.8 | 22.8 | -38.9 | 78.5 | a |
