# Supplementary material for: Metabolite Production in Alkanna tinctoria Links Plant Development with the Recruitment of Individual Members of Microbiome Thriving at the Root-Soil Interface
Source: mSystems. 2022 Sep 7;7(5):e00451-22. doi: 10.1128/msystems.00451-22 (PMC9601132; doi:10.1128/msystems.00451-22)
Supplement: TABLE S3 [file msystems.00451-22-s0008.docx]

| **Metabolite code** | **Compound** | **Chemical formula** | **Molecular weight [g/mol]** | **ID method** | **Retention time [min]** | **Parent ion mass [m/z]** | **Parent ion type** | **Δm [ppm]** | **Matched fragments (rel. int. >10%)** |
| --- | --- | --- | --- | --- | --- | --- | --- | --- | --- |
| M1 | **β, β - Dimethylacrylalkannin** | C_21_H_22_O_6_ | 370.3949 | MS/MS (+) | 9.5 | 393.1288 | [M+Na]^+^ | -5.34 | 392.7, 392.1, 391.4, 375.1, 351.0, 345.5, 344.8, 344.0, 342.7, 341.3, 340.2, 328.4, 293.3, 253.1 |
| M2 | **Isovalerylalkannin** | C_21_H_24_O_6_ | 372.4107 | MS/MS (+) | 9.7 | 395.1443 | [M+Na]^+^ | -5.57 | 409.3, 394.2, 377.2, 345.7, 344.8, 343.8, 343.2, 342.3, 341.1, 340.2, 339.2, 328.7, 327.9, 326.7, 292.9 |
| M3 | **Acetylalkannin** | C_18_H_18_O_6_ | 330.3312 | MS/MS (+) | 8.2 | 353.0988 | [M+Na]^+^ | -2.27 | 352.8, 345.7, 342.4, 335.2, 328.3, 311.1, 293.0, 262.1, 253.0, 183.1, 111.9 |
| M4 | **Alkannin** | C_16_H_16_O_5_ | 288.2946 | MS/MS (+) | 7.4 | 289.1057 | [M+H]^+^ | -4.84 | 271.0 |
| M5 | **Tyrosine** | C_9_H_11_NO_3_ | 181.1881 | MS/MS (+) | 2.6 | 182.0801 | [M+H]^+^ | -6.04 | 164.8, 135.9 |
| M6 | **Rosmarinic acid** | C_18_H_16_O_8_ | 360.3142 | MS/MS (+) | 3.6 | 378.1176 | [M+NH_4_]^+^ | -1.85 | 162.9 |
| M7 | **Phenylalanine** | C_9_H_11_NO_2_ | 165.1887 | MS/MS (+) | 2.6 | 166.0859 | [M+H]^+^ | -2.41 | 119.9 |
| M8 | **Abscisic acid** | C_15_H_20_O_4_ | 264.3161 | MS/MS (+) | 8.8 | 287.1265 | [M+Na]^+^ | 3.83 | 269.0 |
| M9 | **Methyl jasmonate** | C_13_H_20_O_3_ | 224.2953 | MS (+) | 3.3 | 207.1373 | [M-H_2_O+H]^+^ | -3.38 | N/A |
| M10 | **Maltotetraose** | C_24_H_42_O_21_ | 666.5760 | MS/MS (+) | 1.1 | 689.2094 | [M+Na]^+^ | -2.47 | 527.2, 509.2, 365.0, 347.2 |
| M11 | **Raffinose** | C_18_H_32_O_16_ | 504.4358 | MS/MS (+) | 1.1 | 527.1566 | [M+Na]^+^ | -3.22 | 365.1, 347.1 |
| M12 | **6-Methoxyflavanone** | C_16_H_14_O_3_ | 254.2800 | MS/MS (+) | 5.6 | 255.1006 | [M+H]^+^ | -3.92 | 240.1, 237.0, 227.1, 213.0, 211.0, 209.0, 199.1, 185.1, 162.9, 149.9, 104.9 |
| M13 | **Ostruthol** | C_21_H_22_O_7_ | 386.3943 | MS/MS (+) | 7.7 | 369.1329 | [M-H_2_O+H]^+^ | -1.08 | 269.0 |
| M14 | **4-(3,4-Dihydroxyphenyl)-6,7-dihydroxy-2-naphthoic acid** | C_17_H_12_O_6_ | 312.2731 | MS/MS (+) | 3.0 | 313.0699 | [M+H]^+^ | -2.56 | 295.0, 269.0, 251.1 |
| M15 | **Sinapinic acid** | C_11_H_12_O_5_ | 224.2095 | MS/MS (+) | 4.0 | 207.0648 | [M-H_2_O+H]^+^ | -1.93 | 174.9 |
| M16 | **Adenosine** | C_10_H_13_N_5_O_4_ | 267.2408 | MS/MS (+) | 2.2 | 268.1035 | [M+H]^+^ | -1.86 | 135.9 |
| M17 | **4-Hydroxy-3-(3-methylbut-2-en-1-yl)benzoic acid** | C_12_H_14_O_3_ | 206.2372 | MS/MS (+) | 3.8 | 207.1005 | [M+H]^+^ | -5.31 | 150.9 |
| M18 | **2-Amino-1,3,4-octadecanetriol** | C_18_H_39_NO_3_ | 317.5056 | MS/MS (+) | 7.8 | 318.2984 | [M+H]^+^ | -5.97 | 300.2, 282.3 |
| M19 | **Pyrogallol** | C_6_H_6_O_3_ | 126.1098 | MS/MS (+) | 1.2 | 127.0385 | [M+H]^+^ | -3.94 | 108.8, 99.0 |
| M20 | **Caffeic acid** | C_9_H_8_O_4_ | 180.1571 | MS/MS (+) | 3.7 | 181.0489 | [M+H]^+^ | -3.31 | 162.9, 138.9 |
| M21 | **Methyl (7-hydroxy-11-methyl-2,9-dioxo-12,15-dioxatetracyclo[8.4.1.0^1,10^.0^3,8^]pentadeca-3,5,7-trien-13-yl)acetate** | C_17_H_16_O_7_ | 332.3041 | MS/MS (+) | 4.7 | 333.0967 | [M+H]^+^ | -0.60 | 315.0, 297.1 |
| N/A | **12-Oxo-phytodienoic acid** | C_18_H_28_O_3_ | 292.4120 | MS/MS (+) | 7.7 | 293.2105 | [M+H]^+^ | -2.05 | 275.1, 257.2 |
| N/A | **1,3,6,8-Tetrahydroxy-2-[(1S)-1-methoxyhexyl]-9,10-anthraquinone** | C_21_H_22_O_7_ | 386.3943 | MS/MS (+) | 5.7 | 387.1393 | [M+H]^+^ | -11.62 | 355.0 |
| N/A | **1,6,8-trihydroxy-3-(2'-hydroxypropyl)-9,10-anthraquinone** | C_17_H_14_O_6_ | 314.2889 | MS/MS (+) | 5.3 | 297.0746 | [M-H_2_O+H]^+^ | -3.70 | 282.0, 279.0, 269.1, 268.0, 254.1, 251.0, 237.1 |
| N/A | **4-Hydroxybenzoic acid** | C_7_H_6_O_3_ | 138.1205 | MS (+) | 2.4 | 139.0383 | [M+H]^+^ | -5.03 | N/A |
| N/A | **5-(β-D-Glucopyranosyloxy)-2-hydroxybenzoic acid** | C_13_H_16_O_9_ | 316.2601 | MS/MS (-) | 2.5 | 315.0723 | [M-H]^-^ | 0.32 | 164.9, 152.9, 151.9, 108.9, 108.0 |
| N/A | **Citric acid** | C_6_H_8_O_7_ | 192.1232 | MS/MS (-) | 1.1 | 191.0197 | [M-H]^-^ | 0.00 | 172.9, 110.9 |
| N/A | **Citrinin** | C_13_H_14_O_5_ | 250.2467 | MS/MS (+) | 5.8 | 233.0805 | [M-H_2_O+H]^+^ | -1.29 | 218.0, 215.0, 205.1, 190.0, 187.0 |
| N/A | **L-(-)-Malic acid** | C_4_H_6_O_5_ | 134.0872 | MS/MS (-) | 1.1 | 133.0143 | [M-H]^-^ | 0.75 | 114.9 |
| M352T3 | **Senecivernine N-oxide** | C_18_H_25_NO_6_ | 351.1682 | MS/MS (+) | 3.3 | 352.1736 | [M+H]^+^ | -5.29 | 118.0, 120.0, 135.9, 153.9, 254.0 |
| N/A | **Succinic acid** | C_4_H_6_O_4_ | 118.0878 | MS/MS (-) | 1.7 | 117.0192 | [M-H]^-^ | -0.85 | 98.9, 72.9 |
