# Supplementary material for: Metabolite Production in Alkanna tinctoria Links Plant Development with the Recruitment of Individual Members of Microbiome Thriving at the Root-Soil Interface
Source: mSystems. 2022 Sep 7;7(5):e00451-22. doi: 10.1128/msystems.00451-22 (PMC9601132; doi:10.1128/msystems.00451-22)
Supplement: TABLE S4 [file msystems.00451-22-s0009.docx]

| **Absolute abundance values of Bacterial SDA-rASVs** | | | | | | | | | |
| --- | --- | --- | --- | --- | --- | --- | --- | --- | --- |
| **ASV 223** | *Allorhizobium-N.-P.-Rhizobium* | | | | **ASV 187** | *Phenylobacterium* | | | |
| **Root** | **growth** | **blooming** | **fruiting** | **decay** | **Root** | **growth** | **blooming** | **fruiting** | **decay** |
| Austrian | 0 | 0 | 0 | 0 | Austrian | 0 | 12 | 0 | 1287 |
| Greek A | 39 | 161 | 295 | 384 | Greek A | 0 | 0 | 0 | 188 |
| Greek B | 72 | 80 | 48 | 90 | Greek B | 0 | 0 | 0 | 125 |
| **ASV 194** | *Allorhizobium-N.-P.-Rhizobium* | | | | **ASV 37** | *Burkholderia-C.-Paraburkholderia* | | | |
| **Root** | **growth** | **blooming** | **fruiting** | **decay** | **Root** | **growth** | **blooming** | **fruiting** | **decay** |
| Austrian | 178 | 148 | 193 | 574 | Austrian | 0 | 93 | 0 | 327 |
| Greek A | 0 | 0 | 22 | 0 | Greek A | 748 | 1102 | 400 | 548 |
| Greek B | 0 | 0 | 0 | 0 | Greek B | 560 | 534 | 748 | 366 |
| **Rhizosphere** | **growth** | **blooming** | **fruiting** | **decay** | **Rhizosphere** | **growth** | **blooming** | **fruiting** | **decay** |
| Austrian | 290 | 310 | 351 | 379 | Austrian | 0 | 74 | 20 | 3119 |
| Greek A | 0 | 0 | 31 | 0 | Greek A | 795 | 1056 | 570 | 1121 |
| Greek B | 0 | 0 | 0 | 0 | Greek B | 661 | 349 | 599 | 1166 |
| **Soil** | **growth** | **blooming** | **fruiting** | **decay** | **Soil** | **growth** | **blooming** | **fruiting** | **decay** |
| Austrian | 677 | 467 | 907 | 510 | Austrian | 0 | 233 | 216 | 104 |
| Greek A | 0 | 0 | 250 | 0 | Greek A | 1478 | 1662 | 907 | 495 |
| Greek B | 0 | 0 | 189 | 0 | Greek B | 1559 | 1454 | 1561 | 594 |
| **ASV 1231** | *Burkholderia-C.-Paraburkholderia* | | | | **ASV 625** | *Mucilaginibacter* | | | |
| **Root** | **growth** | **blooming** | **fruiting** | **decay** | **Root** | **growth** | **blooming** | **fruiting** | **decay** |
| Austrian | 0 | 20 | 20 | 0 | Austrian | 0 | 0 | 0 | 0 |
| Greek A | 62 | 159 | 67 | 5 | Greek A | 195 | 153 | 28 | 55 |
| Greek B | 0 | 0 | 0 | 0 | Greek B | 0 | 0 | 0 | 9 |
| **ASV 553** | *Gammaproteobacteria* | | | | **Soil** | **growth** | **blooming** | **fruiting** | **decay** |
| **Root** | **growth** | **blooming** | **fruiting** | **decay** | Austrian | 0 | 32 | 49 | 0 |
| Austrian | 0 | 0 | 0 | 367 | Greek A | 98 | 297 | 44 | 56 |
| Greek A | 0 | 0 | 0 | 192 | Greek B | 0 | 0 | 0 | 0 |
| Greek B | 0 | 0 | 0 | 49 | **Absolute abundance values of Fungal SDA-rASVs** | | | | |
| **Rhizosphere** | **growth** | **blooming** | **fruiting** | **decay** | **ASV 41** | *Penicillium jensenii* | | | |
| Austrian | 0 | 0 | 0 | 45 | **Root** | **growth** | **blooming** | **fruiting** | **decay** |
| Greek A | 0 | 0 | 0 | 120 | Austrian | 0 | 0 | 455 | 176 |
| Greek B | 0 | 0 | 0 | 295 | Greek A | 0 | 0 | 920 | 277 |
| **Soil** | **growth** | **blooming** | **fruiting** | **decay** | Greek B | 277 | 1077 | 21272 | 1638 |
| Austrian | 0 | 0 | 0 | 18 | **ASV 122** | *Helotiaceae* | | | |
| Greek A | 0 | 8 | 34 | 250 | **Root** | **growth** | **blooming** | **fruiting** | **decay** |
| Greek B | 3 | 0 | 0 | 281 | Austrian | 63 | 7 | 0 | 99 |
|  |  |  |  |  | Greek A | 318 | 20 | 0 | 0 |
|  |  |  |  |  | Greek B | 939 | 1344 | 0 | 390 |
