# Supplementary material for: Metabolite Production in Alkanna tinctoria Links Plant Development with the Recruitment of Individual Members of Microbiome Thriving at the Root-Soil Interface
Source: mSystems. 2022 Sep 7;7(5):e00451-22. doi: 10.1128/msystems.00451-22 (PMC9601132; doi:10.1128/msystems.00451-22)
Supplement: TABLE S5 [file msystems.00451-22-s0010.docx]

| **Soil name** | **Location** | **Altitude (m)** | **Climate** | **pH** | **Conductivity (µS/cm)** | **Ca** | **Fe** | **K** | **Mg** | **Mn** | **P** |
| --- | --- | --- | --- | --- | --- | --- | --- | --- | --- | --- | --- |
| Austrian | 48°08'38" 16°22'17.0" | 186 | Temperate-pannonic | 7.91 | 153.3 | 347 | 1.9 | 11.6 | 9.5 | 6.1 | 0.57 |
| Greek A | 40°64’  22°96’ | 50-70 | Mediterranean | 7.81 | 116.0 | 97 | 1.6 | 1.0 | 6.1 | 2.1 | 0.20 |
| Greek B | 40°63’  22°97’ | 130 | Mediterranean | 7.13 | 105.8 | 86 | 2.4 | 1.2 | 4.4 | 3.3 | 0.35 |
